# Splenic hilar lymph node dissection enhances survival in Bormann type 4 gastric cancer

**DOI:** 10.1038/s41598-023-42707-9

**Published:** 2023-09-16

**Authors:** Oh Jeong, Han Hong Lee, Hoon Hur, Hyoung-Il Kim

**Affiliations:** 1 Division of Gastroenterological Surgery, Department of Surgery, Chonnam National University School of Medicine, Gwangju, South Korea; 2 Department of Surgery, Seoul St. Mary’s Hospital, College of Medicine, The Catholic University of Korea, Seoul, Korea; 3 Department of Surgery, Ajou University School of Medicine, Suwon, Korea; 4 Department of Surgery, Yonsei University College of Medicine, 50-1 Yonsei-Ro Seodaemun-Gu, Seoul, 03722 Republic of Korea

**Keywords:** Gastric cancer, Surgical oncology

## Abstract

Splenic hilar (no.10) lymph node dissection during total gastrectomy is no longer recommended for advanced proximal gastric cancer. However, the treatment efficacy of no.10 lymph node dissection in Borrmann type 4 tumors remains unclear. We enrolled 539 patients who underwent total gastrectomy for Borrmann type 4 tumors between 2006 and 2016 in four major institutions in Korea. We compared the long-term survival of the no.10 lymph node dissection (n = 309) and no-dissection groups (n = 230) using the propensity score (inverse probability of treatment weighting). The treatment effects of no.10 lymph node dissection were estimated in the weighted sample using the Cox proportional hazards regression model with a robust sandwich-type variance estimator. After inverse probability of treatment weighting, there were 540.4 patients in the no.10 lymph node dissection group and 532.7 in the no-dissection group. The two groups showed well-balanced baseline characteristics, including tumor node metastasis stage. The 5-year survival rates in the no.10 lymph node dissection and no-dissection groups were 45.7% and 38.6%, respectively (log-rank p = 0.036, hazard ratio 0.786, 95% confidence interval 0.630–0.982). Multivariate analysis revealed that no.10 lymph node dissection was an independent favorable prognostic factor (adjusted hazard ratio 0.747, 95% confidence interval 0.593–0.940) after adjusting for other prognostic factors. Sensitivity analyses in other inverse probability of treatment weighting models and the propensity score matching model showed similar results. Patients undergoing no.10 lymph node dissection showed improved survival compared to those without. No.10 lymph node dissection is recommended during total gastrectomy for patients with Borrmann type 4 gastric cancer.

## Introduction

Although the global incidence of gastric cancer has declined over recent decades, the incidence of proximal gastric cancer has gradually increased in Korea and Japan^1,2^. Total gastrectomy with regional lymph node dissection (LND) is a standard surgery for proximal gastric cancer. The lymph nodes around the splenic hilum (no.10 LNs) are regional lymph nodes that should be removed in D2 LND^3^. However, prophylactic splenectomy to remove no.10 LNs is no longer recommended for proximal gastric cancer because it does not improve treatment outcomes when combined with total gastrectomy compared with total gastrectomy alone^4,5^. Nonetheless, the trial that presented this result excluded patients with proximal gastric cancer invading the greater curvature or Borrmann type 4 (B-4) tumors. Thus, the necessity of no.10 LND in those patients remains uncertain.

B-4 tumors, also known as scirrhous gastric cancer, are an uncommon type characterized by diffuse thickening and sclerosis of the gastric wall without marked ulceration or a raised margin. Previous studies found that B-4 tumors were a main factor in increased risk for no.10 LN metastasis, with the incidence ranging from 15 to 26%^6,7^. Therefore, many surgeons still believe that no.10 LND may have a local control effect when treating B-4 tumors^8–10^. Few studies have investigated the efficacy of no.10 LND in B-4 tumors, and those that have were limited by their focus on the therapeutic index^8–10^ or small case number^11^.

Therefore, the efficacy of no.10 LND in B-4 tumors should be examined in a large cohort. In this study, to determine the survival effect of no.10 LND in B-4 tumors, we compared the long-term survival of patients with and without no.10 LND in a large multi-institutional dataset.

## Methods

### Patients

**Figure 1:**
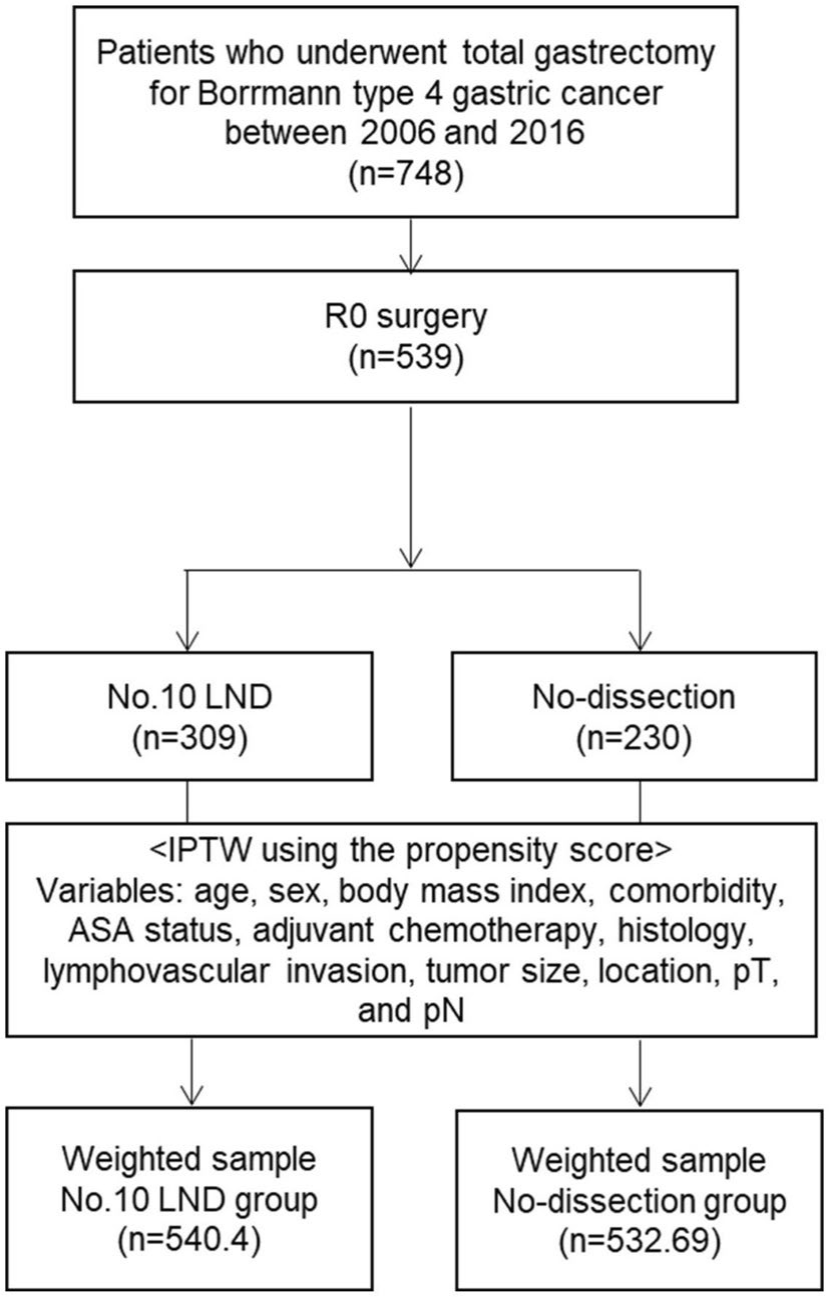
Flow diagram. *No.10 LND* splenic hilar lymph node dissection, *IPTW* inverse probability of treatment weighting, *ASA status* American Society of Anesthesiologists physical status.

The present study included patients who underwent total gastrectomy for B-4 tumors between 2006 and 2016 in four major institutions in South Korea (Ajou University, Chonnam National University, Seoul St. Mary’s Hospital, and Yonsei University). B-4 tumors were defined as diffuse, infiltrative tumors with thickened and indurated gastric walls without marked ulceration or raised margins. We included patients based on macroscopic findings in pathologic reports and specimen photos, as available. Patients were excluded if they received preoperative chemotherapy, had a history of concomitant malignant diseases, or had incomplete medical records. We identified 748 patients who met the eligibility criteria and finally included 539 patients who underwent curative (R0) surgery (Fig. 1). We divided patients into two groups (no.10 LND group vs. no-dissection group) and compared their long-term survival. The institutional review board at each institution approved this study (Institutional Review Board of Ajou University Hospital: MDB-2021-355; Chonnam National Universtiy Hospital Biomedical Research Ethics Committee: CNUHH-2021-174; The institutional review board of the ethics committee of the College of Medicine, the Catholic University of Korea: KC23RIDI0411; and Yonsei University Health System, Severance Hospital, Institutional Review Board: 4-2022-1397), and the requirement for informed consent was waived. All methods were performed in accordance with the relevant guidelines and regulations.

### Operative techniques

Patients underwent total gastrectomy and regional LND as described in the gastric cancer treatment guidelines^12^ and received D1/D1+ or D2 LND at the discretions of surgeons. D2 LND included nos.7 (left gastric), 8a (common hepatic), 9 (celiac), 12a (proper hepatic), 11p (proximal splenic), 11d (distal splenic), and 10 (splenic hilum) LNs in addition to the perigastric LNs. D1 + LND included only nos.7, 8a, 9, and 11p. Removal of no.10 LNs was carried out via splenectomy, the spleen-preserving technique, or distal pancreaticosplenectomy. As this study adopted a retrospective multicenter design, a standardized protocol for performing no.10 LND was not established. Generally, surgeons tended to undertake no.10 LND in cases presenting with more advanced tumors. As for the operative technique, the principle technique for no.10 LND was the spleen preserving technique. Nonetheless, when LN metastasis was highly suspected at the splenic hilum, the decision to perform splenectomy was contingent upon surgeons’ discretion. All patients received esophagojejunostomy after total gastrectomy.

Patients underwent postoperative follow-ups every 6 months for 5 years. Abdominal computed tomography (CT) and endoscopy were routinely performed for surveillance every 6 or 12 months. Other imaging tests, such as chest CT, liver magnetic resonance imaging (MRI), and positron emission tomography (PET)/CT scan, were performed as appropriate. Patients with pathologic stage II or higher received adjuvant chemotherapy using S-1 or capecitabine plus oxaliplatin.

### Data collection

We collected clinicopathological data using a standardized case report form, which included demographics (age, sex, body mass index, comorbidity, American Society of Anesthesiologists [ASA] physical status, and past medical history), preoperative tests (CT and endoscopy findings, tumor markers, and laboratory data), operative outcomes (curability, operative techniques, operating time, operative bleeding, and extent of LND), pathological results (histologic type, lymphovascular invasion, tumor size, resection margin, tumor location, number of harvested and metastatic LNs, and tumor node metastasis [TNM] stage), postoperative outcomes (diet start, hospital stay, and complications), and follow-up data (adjuvant chemotherapy, disease recurrence, and survival).

The tumor characteristics and operative techniques were recorded based on the third English edition of the Japanese Classification of Gastric Carcinoma^13^. The pathologic stage was based on the eighth edition of the Union for International Cancer Control TNM classification of gastric carcinoma^14^. Curative (R0) surgery was defined as macroscopic and microscopic complete tumor removal without distant metastasis. Postoperative complications were defined as any complications that developed within 30 days of surgery. Overall survival was defined as the time from operation until death by any cause or last follow-up. Survival was ascertained using patient medical records or national cancer registry data. The last follow-up was in December 2021, and the median follow-up duration was 37 months (range 1–152 months).

### Inverse probability of treatment weighting using propensity score

The propensity score was calculated with a logistic regression model incorporating 12 variables: age, sex, body mass index, comorbidity, ASA status, adjuvant chemotherapy, histologic type, lymphovascular invasion, tumor size, tumor location, pT, and pN stage. We then performed inverse probability of treatment weighting (IPTW) using the propensity score. IPTW is preferable to matching when the size of the control group is insufficient, as in our study^15^, as it enables estimation of the average treatment effect (ATE) using the overall sample as the reference population. In this study, the IPTW weights were defined as ${\omega }_{\iota }\text{=}\frac{{Z}_{{i}}}{{e}_{{i}}}+\frac{\left(1-{Z}_{{i}}\right)}{1-{e}_{{i}}}$ (where Z_*i*_ is an indicator variable of whether the *i*th subject was treated, and *e*_*i*_ denotes the propensity score for the *i*th subject), as proposed by Rosenbaum et al.^16^ After IPTW, the analysis set comprised 540.4 patients in the no.10 LND group and 532.69 patients in the no-dissection group (Fig. 1).

### Statistics

We expressed data as mean ± standard deviation or number (%). Continuous variables were compared using the *t*-test, and categorical variables using the chi-square test. We calculated weighted Kaplan–Meier estimates for survival curves in the IPTW sample. We used a modified log-rank test appropriate for use with a weighted sample to compare survival. The causal effects of treatment (no.10 LND) were estimated using the Cox proportional hazards regression model. A robust, sandwich-type variance estimator was used to account for the weighted nature of the sample.

As a sensitivity analysis, we tested the treatment effect of no.10 LND in other IPTW models using different weights, such as the average treatment effects on the treated (ATT) and stabilized ATE weights, and in the propensity score matching sample. Statistical analysis was performed using SPSS 21.0 (IBM Corp., NY, USA) and R (version 4.1.2, Vienna, Austria). Two-sided p-values less than 0.05 were considered statistically significant.

## Results

### Patient characteristics

The original sample consisted of 302 men and 237 women with a mean age of 57.2 ± 13.2 years (Table 1). A total of 309 patients received no.10 LND, and 230 patients did not. Of those who underwent no.10 LND, 227 received the spleen-preserving technique, 76 underwent splenectomy, 6 underwent distal pancreaticosplenectomy, and 19.1% (59 of 309 patients) showed metastasis to no.10 LNs. The original sample showed significant differences in ASA score (p = 0.039), tumor size (p < 0.001), tumor location (p = 0.001), tumor depth (p = 0.049), and TNM stage (p = 0.014) between the two groups. The no.10 LND group was associated with a larger tumor size and more advanced TNM stage compared with the no-dissection group. Tumors involving the whole stomach were more frequent in the no.10 LND group.

**Table 1:** Baseline characteristics in the original and IPTW samples.

Variables	Original sample				IPTW sample			
	No.10 LND		P	SMD	No.10 LND		P	SMD
	Not performed (n = 230)	Performed (n = 309)			Not performed (n = 532.69)	Performed (n = 540.40)		
Age (years)	58.2 ± 13.9	56.5 ± 12.8	0.142	0.126	57.3 ± 13.9	57.2 ± 12.7	0.123	0.009
Sex			0.468	0.063			0.933	0.005
Male	133 (57.8)	169 (54.7)			295.2 (55.4)	300.8 (55.7)		
Female	97 (42.2)	140 (45.3)			237.5 (44.6)	239.6 (44.3)		
BMI (kg/m^2^)	22.6 ± 3.3	22.5 ± 2.9	0.709	0.048	22.5 ± 3.4	22.5 ± 2.8	1.000	0.008
Comorbidity	112 (48.7)	148 (47.9)	0.854	0.016	249.2 (46.8)	257.4 (47.6)	0.780	0.017
ASA status			0.039	0.224			0.502	0.072
1	129 (56.1)	139 (45.0)			273.0 (51.3)	265.8 (49.2)		
2	87 (37.8)	147 (47.6)			219.5 (41.2)	240.0 (44.4)		
≥ 3	14 (6.1)	23 (7.4)			40.1 (7.5)	34.5 (6.4)		
Chemotherapy	172 (74.8)	251 (81.2)	0.058	0.156	417.0 (78.3)	426.0 (78.8)	0.808	0.013
Histology			0.067	0.158			0.781	0.016
Differentiated	26 (11.3)	21 (6.8)			47.9 (9.0)	46.0 (8.5)		
Undifferentiated	204 (88.7)	288 (93.2)			484.8 (91.0)	494.4 (91.5)		
Lymphovascular invasion	149 (64.8)	193 (62.5)	0.579	0.048	338.8 (63.6)	342.1 (63.3)	0.920	0.006
Tumor size (cm)	9.4 ± 4.6	10.9 ± 5.1	< 0.001	0.307	10.1 ± 4.8	10.2 ± 4.9	0.730	0.025
Tumor location			0.001	0.351			0.908	0.028
Lower	52 (22.6)	42 (13.6)			93.6 (17.6)	94.0 (17.4)		
Middle	83 (36.1)	111 (35.9)			197.5 (37.1)	195.0 (36.1)		
Upper	74 (32.2)	96 (31.1)			66.1 (31.2)	170.6 (31.6)		
Whole stomach	21 (9.1)	60 (19.4)			75.4 (14.2)	80.8 (15.0)		
T stage^a^			0.046	0.244			0.999	0.009
T2	18 (7.8)	12 (3.9)			31.5 (5.9)	32.5 (6.0)		
T3	40 (17.4)	37 (12.0)			75.6 (14.2)	75.2 (13.9)		
T4a	166 (72.2)	249 (80.6)			408.6 (76.7)	415.3 (76.8)		
T4b	6 (2.6)	11 (3.6)			17.0 (3.2)	17.4 (3.2)		
N stage^a^			0.518	0.157			0.997	0.023
N0	42 (18.3)	68 (22.0)			101.8 (19.1)	106.3 (19.7)		
N1	24 (10.4)	26 (8.4)			46.7 (8.8)	48.6 (9.0)		
N2	38 (16.5)	50 (16.2)			89.3 (16.8)	91.4 (16.9)		
N3a	60 (26.1)	66 (21.4)			134.7 (25.3)	132.0 (24.4)		
N3b	66 (28.7)	99 (32.0)			160.2 (30.1)	162.1 (30.0)		
TNM stage^a^			0.014	0.325			0.080	0.132
Ib	9 (3.9)	3 (1.0)			16.3 (3.1)	8.9 (1.7)		
IIa	18 (7.8)	11 (3.6)			34.3 (6.4)	21.3 (3.9)		
IIb	28 (12.2)	60 (19.4)			72.6 (13.6)	92.8 (17.2)		
IIIa	45 (19.6)	61 (19.7)			101.1 (19.0)	112.0 (20.7)		
IIIb	54 (23.5)	67 (21.7)			119.4 (22.4)	133.8 (24.8)		
IIIc	76 (33.0)	107 (34.6)			189.0 (35.5)	171.6 (31.8)		

Data are expressed as mean ± standard deviation or n (%).

^a^TNM stage is based on the eighth edition AJCC TNM classification of gastric carcinoma.

*IPTW* inverted probability of treatment weighting, *No.10 LND* splenic hilar lymph node dissection, *BMI* body mass index, *ASA status* American Society of Anesthesiologists physical status, *SMD* standardized mean difference, *TNM* tumor node metastasis.

In the IPTW sample, there were 540.4 patients in the no.10 LND group and 532.7 patients in the no-dissection group (Table 1). After IPTW, the two groups showed well-balanced baseline characteristics: the standardized mean differences of 12 matching covariates were all below 0.2 (range 0.005–0.072). There were no statistically significant differences in the groups’ clinicopathological characteristics, including the T and N stages.

### Short-term outcomes

We analyzed short-term surgical outcomes in the original sample to investigate the crude effects of no.10 LND on surgical outcomes (Table 2). The overall morbidity in the no.10 LND and no-dissection groups was 60.5% and 51.9%, respectively (p = 0.047). However, there were no significant differences in mortality (1.3% vs. 1.7%, p = 0.729), ≥ grade 3 complications (34.6% vs. 34.6%, p = 0.999), or mean hospital stay (10.3 days vs. 10.8 days, p = 0.399) between the two groups.

**Table 2:** Operative outcomes in the original sample.

	No.10 LND		P
	Performed (n = 309)	Not performed (n = 230)	
Operative approach			0.367
Open	272 (88.0)	208 (90.5)	
Laparoscopy	37 (12.0)	22 (9.5)	
Operating time (min)	215 ± 58	213 ± 63	0.645
Operative bleeding (ml)	225 ± 327	193 ± 196	0.220
Overall morbidity	187 (60.5)	120 (51.9)	0.047
Mortality	4 (1.3)	4 (1.7)	0.729
≥ Grade 3 morbidity	107 (34.6)	80 (34.6)	0.999
Complication types			
Local complications	51 (16.5)	46 (19.9)	0.307
Systemic complications	63 (20.4)	34 (14.7)	0.089
Both	73 (23.6)	40 (17.3)	0.074
Hospital stay	10.3 ± 7.1	10.8 ± 6.5	0.399

Data are expressed as mean ± standard deviation or n (%).

*No.10 LND* splenic hilar lymph node dissection.

### Survival analysis

In the original sample, the two groups showed no significant difference in overall survival (Supplementary Fig. 1). The 5-year survival rates (5-ysr) in the no.10 LND and no-dissection groups were 42.1% and 40.2%, respectively (log-rank p = 0.609). However, in the TNM stage subgroups, the no.10 LND group showed significantly better survival than the no-dissection group in stage IIIa (5-ysr, 57.6% vs. 50.7%, p = 0.014) (Supplementary Fig. 2). The no.10 LND group also showed better survival in stage IIIb (5-ysr, 45.0% vs. 31.1%), but it was not statistically significant (p = 0.179).

**Figure 2:**
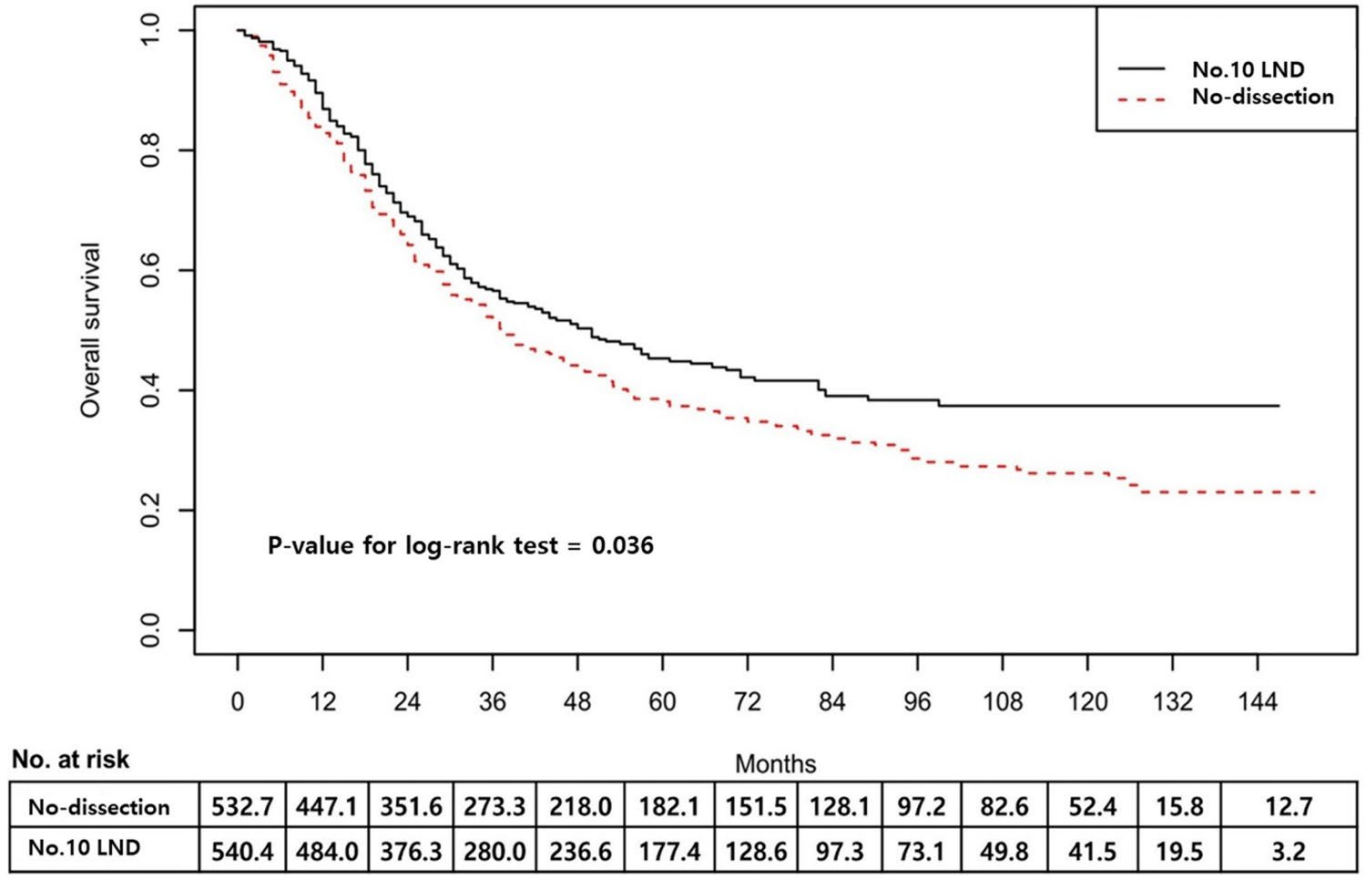
Survival curves in the IPTW sample. *IPTW* inverse probability of treatment weighting, *No.10 LND* splenic hilar lymph node dissection.

**Figure 3:**
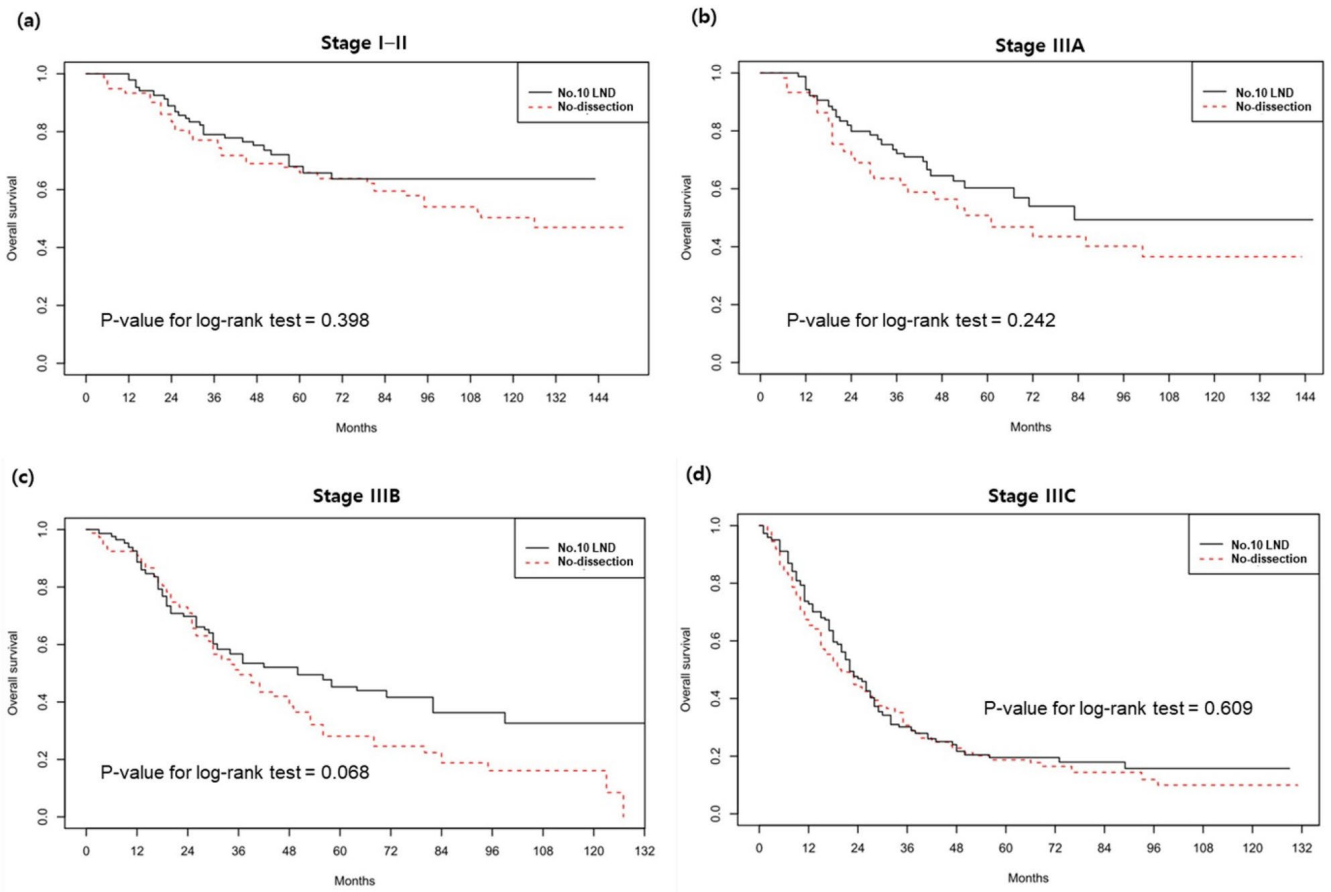
Survival by stage subgroup in the IPTW sample. (**a**) Stages I–II, (**b**) stage IIIa, (**c**) stage IIIb, and (**d**) stage IIIc. *No.10 LND* splenic hilar lymph node dissection.

In the IPTW sample, the no.10 LND group showed significantly better survival than the no-dissection group (Fig. 2). The 5-ysr of the no.10 LND group and no-dissection group were 45.7% and 38.6%, respectively (log-rank p = 0.036). The hazard ratio of no.10 LND was 0.786 (95% CI 0.630–0.982). Figure 3 shows the survival curves in the TNM stage subgroups. The survival difference was most noticeable in the stage IIIa (5-ysr, 60.7% vs. 51.7%, p = 0.242) and stage IIIb groups (5-ysr, 46.0% vs. 29.2%, p = 0.068), although they did not reach statistical significance.

Table 3 presents the univariate and multivariate analyses of prognostic factors in the IPTW sample. Besides no.10 LND, age, ASA status, tumor size, lymphovascular invasion, tumor location, T stage, and N stage were significant prognostic factors in the univariate analysis. Multivariate analysis of these factors revealed that no.10 LND was a favorable prognostic factor (adjusted hazard ratio 0.747, 95% CI 0.593–0.940) after adjusting for other prognostic factors.

**Table 3:** Univariate and multivariate analyses of prognostic factors in the IPTW sample.

	Univariate			Multivariate		
	HR	95% CI	P	aHR	95% CI	P
No.10 LND	0.786	0.630–0.982	0.012	0.747	0.593–0.940	0.010
Age (years)	1.018	1.008–1.028	< 0.001	1.011	1.001–1.021	0.015
Sex (male)	1.045	0.833–1.310	0.701			
Comorbidity	1.044	0.835–1.306	0.700			
ASA status	1.317	1.077–1.612	0.007	1.148	0.927–1.423	0.144
Adjuvant chemotherapy	1.079	0.806–1.444	0.610			
Histology (undifferentiated)	1.388	0.895–2.151	0.142			
Tumor size (cm)	1.095	1.072–1.120	< 0.001	1.057	1.028–1.086	< 0.001
Lymphovascular invasion	1.438	1.135–1.821	0.003	1.051	0.804–1.375	0.805
Tumor location (vs. lower)						
Middle	0.787	0.580–1.068	0.124	0.924	0.655–1.304	0.624
Upper	0.673	0.491–0.921	0.014	0.746	0.525–1.062	0.091
Whole	1.634	1.173–2.277	0.004	1.265	0.865–1.849	0.289
T stage (vs. T2)						
T3	1.310	0.708–2.424	0.390	1.321	0.710–2.459	0.261
T4a	2.290	1.320–3.974	0.003	1.644	0.943–2.864	0.060
T4b	4.212	2.098–8.456	< 0.001	3.611	1.689–7.721	< 0.001
N stage (vs. N0)						
N1	0.955	0.557–1.635	0.865	0.837	0.485–1.448	0.420
N2	1.599	1.040–2.458	0.032	1.241	0.808–1.906	0.397
N3a	2.456	1.698–3.554	< 0.001	1.674	1.144–2.449	0.014
N3b	3.864	2.670–5.592	< 0.001	2.733	1.817–4.111	< 0.001

*HR* hazard ratio, *CI* confidence interval, *No.10 LND* splenic hilar lymph node dissection, *ASA status* American Society of Anesthesiologists physical status.

### Sensitivity analysis

We estimated the survival effects of the no.10 LND in the different IPTW models and the propensity score matching model (Table 4). The estimated hazard ratios were 0.799 (95% CI 0.638–0.998) in the ATT-weighted sample and 0.785 (95% CI 0.629–0.980) in the stabilized ATE-weighed sample. The propensity score matching sample also showed a significant survival benefit for no.10 LND (hazard ratio 0.766, 95% CI 0.597–0.983).

**Table 4:** Survival effects of no.10 LND in the various propensity score models.

	Hazard ratio	95% confidence interval	
		Lower	Upper
IPTW samples using different weights			
ATE weight	0.786	0.630	0.982
Stabilized ATE weight	0.785	0.629	0.980
ATT weight	0.799	0.638	0.998
PSM sample	0.766	0.597	0.983

*IPTW* inverted probability of treatment weighting, *ATE* average treatment effect, *ATT* average treatment effect for the treated, *PSM* propensity score matching, *No.10 LND* splenic hilar lymph node dissection.

## Discussion

This is the first study to compare the long-term survival of B-4 tumor patients who underwent total gastrectomy with and without no.10 LND. We collected the largest existing sample of B-4 tumor patients from four major gastric cancer clinics in Korea. Furthermore, we adopted IPTW using the propensity score to generate robust analysis results considering an insufficient number of the control group. The survival benefit of no.10 LND shown in this study encourages its addition to total gastrectomy for B-4 gastric cancer.

Many invasive procedures have been discouraged in gastric cancer surgery in recent decades, including distal pancreaticosplenectomy to remove LNs along the splenic artery^17,18^, the thoracoabdominal approach for cardia or subcardia cancer^19^, para-aortic LND as an addition to D2 lymphadenectomy^20^, and palliative gastrectomy as an addition to chemotherapy^21^. Similarly, the JCOG-0110 trial demonstrated the non-inferiority of total gastrectomy alone versus with splenectomy^4^. However, the JCOG-0110 trial did not include patients with proximal cancer invading the greater curvature or B-4 tumors, which carry a higher incidence of no.10 LN metastasis, thus necessitating the removal of no.10 LNs. Nevertheless, there is little evidence to support the efficacy of no.10 LND in these tumors.

Kano et al.^9^ insisted on the efficacy of no.10 LND after determining the therapeutic index of no.10 LNs in B-4 tumors, which was similar to that of other perigastric LNs. Furthermore, Hayashi et al.^8^ analyzed the therapeutic index of each LN station in B-4 tumors and found that the incidence of no.10 LN metastasis was 15% and the therapeutic index of no.10 LNs exceeded that of other regional LNs. However, the therapeutic index is an indirect measure of treatment efficacy, which is a simple combination of the incidence of LN metastasis and survival. Only one study directly compared the long-term survival of patients with B-4 or proximal cancer invading the greater curvature who underwent no.10 LND (splenectomy) versus those who did not undergo dissection (spleen preservation)^11^. Unlike our results, they showed that splenectomy did not prolong survival, but they included only 44 B-4 tumor patients. Moreover, they did not differentiate between the greater curvature invading tumors and B-4 tumors. In contrast, our study exclusively concentrated on B-4 tumors, encompassing a substantial cohort of 539 patients. We believe this probably contributed to the disparate findings.

In the present study, the original sample did not show a difference in survival between the no.10 LND group and the no-dissection group. However, it should be noted that the TNM stage was significantly different between the two groups, i.e., the no.10 LND group had a more advanced stage on average. In subgroup analysis, the no.10 LND group showed significantly and marginally better survival in stages IIIa and IIIb, respectively. After the IPTW balanced the tumor stage and other clinicopathological characteristics between the two groups, the no.10 LND group showed significantly better survival. The improved survival was most noticeable in the stage IIIa and IIIb groups in the IPTW sample, although it was not statistically significant due to the small sample size. We performed sensitivity analyses using other IPTW models and a matching model to ensure robust analyses. The conventional ATT weight model can be biased due to over- or underestimating the subjects with very low or high propensity scores. To address this instability, stabilized ATE weights or ATT weights offer a good alternative. Stabilized weights are obtained by multiplying the ATE weight by the probability of receiving the treatment, and ATE weights are calculated using the treated subject as the reference population^15^. As a result, all models showed similar results for the treatment effect of no.10 LND.

One of the main drawbacks of no.10 LND is operative difficulties and related complications. No.10 LND is technically challenging because of the deep anatomic location and poor visualization. Many retrospective studies adopted an open approach and reported higher morbidity rates but equivalent or slightly worse survival in the splenectomy group^22–24^. Meanwhile, the augmented operating view and intra-abdominal approach afforded by laparoscopic surgery have enabled meticulous and precise skeletonization of the complex vascular structure. Recent studies have reported acceptable morbidity rates of spleen-preserving no.10 LND using laparoscopy^25,26^. Our study showed slightly higher overall morbidity in the no.10 LND group but no significant increase in mortality or ≥ grade 3 complications. We believe that the safety and feasibility of no.10 LND are adequate if the procedure is properly performed by experienced surgeons.

No.10 LND can be performed in various ways, such as splenectomy, spleen preserving technique, or distal pancreatectomy. In our study, the spleen-preserving technique was most frequently performed for no.10 LND. This technique, in which the splenic vessels at the hilum are skeletonized to remove the no.10 LNs, has become widely used in Korea. One small randomized controlled trial demonstrated no significant survival difference between the spleen-preserving technique and splenectomy in proximal gastric cancer^27^. Many retrospective studies have also reported no survival difference between the splenectomy and spleen-preserving technique^28–30^. The spleen-preserving technique may be a good alternative for removing no.10 LNs, as it avoids splenectomy-associated complications. However, our study did not differentiate between the two no.10 LND techniques (spleen preserving technique and splenectomy), leaving uncertainty regarding their comparative treatment effect in B-4 tumors. Additionally, potential bias associated with the diverse techniques employed for no.10 LND in this study should be acknowledged when interpreting our findings.

No.10 LND may not offer advantages to all patients with B-4 tumors. In the subgroup analysis, the disparity in survival did not reach statistical significance across all subgroups. Nevertheless, a noticeable difference was observed within the subgroups of stage IIIa and IIIb. The survival rates were nearly similar within stage IIIc. While this study refrained from offering a definitive explanation about this observation, it is conceivable that the poor prognosis with stage IIIc remains largely unaffected by performing no.10 LND. Furthermore, in theory, the potential benefits of no.10 LND are likely limited to patients exhibiting LN metastasis at the splenic hilum. Unfortunately, existing imaging techniques lack the precision required to accurately diagnose LN metastasis at the splenic hilum before surgery. Instead, we can potentially rely on clinical indicators, such as T and N factors, to predict the presence of splenic LN metastasis and determine the appropriateness of no.10 LND. This concern presents an additional challenge that must be addressed before the incorporation of no.10 LND into clinical practice for B4-type tumors. To address this matter, we are in the process of gathering more comprehensive clinical data to facilitate a future study aimed at resolving these issues.

This study has some limitations. First, although we performed IPTW to control the effect of confounders, selection bias could not be completely ruled out. Because of the retrospective nature of this study, surgeons may have preferred no.10 LND for patients who had a large tumor or more advanced TNM stage. Second, different extents of nodal dissection may cause stage migration, which could lead to an inappropriate comparison of the TNM stage between the two groups. In our data, however, stage migration attributed to adding no.10 dissection was minimal in the no.10 LND group (only 3 of 230 patients), and this may not significantly affect our results. Third, we did not collect data about tumor recurrence and disease-free survival in this study. Thus, future studies should further investigate the difference in recurrence patterns between the two groups. Lastly, various methods were used for no.10 LND in our study’s subjects, so an appropriate technique cannot be recommended based on our results.

In conclusion, this multi-institutional study on B-4 gastric cancer demonstrated that patients who underwent no.10 LND showed better survival than the no-dissection group. No.10 LND slightly increased the operative morbidity but not severe morbidity or mortality. Thus, dissection of no.10 lymph nodes during total gastrectomy is recommended for patients with B-4 gastric cancer.

### Supplementary Information

Supplementary Figures.

## Data Availability

The datasets used and analyzed during the current study available from the corresponding author on reasonable request.
