# Supplementary material for: Splenic hilar lymph node dissection enhances survival in Bormann type 4 gastric cancer
Source: Sci Rep. 2023 Sep 16;13:15390. doi: 10.1038/s41598-023-42707-9 (PMC10505185; doi:10.1038/s41598-023-42707-9)

Supplementary Fig. 1 Survival curves in the no.10 LND group and the no-dissection group in the original sample


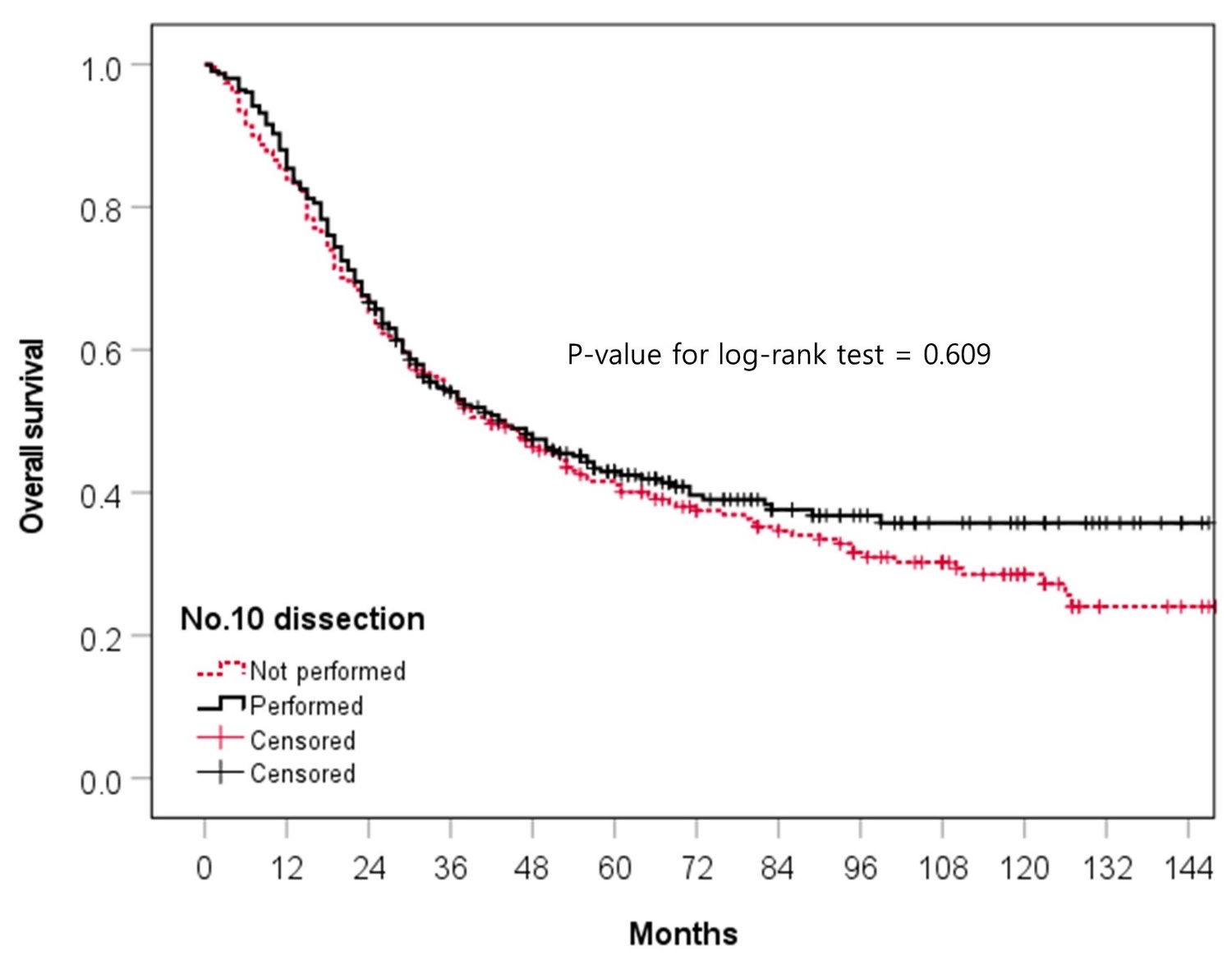


Supplementary Fig. 2 Survival curves in the subgroups by TNM stage in the original sample: (a) stages I–II, (b) stage IIIa, (c) stage IIIb, and (d) stage IIIc


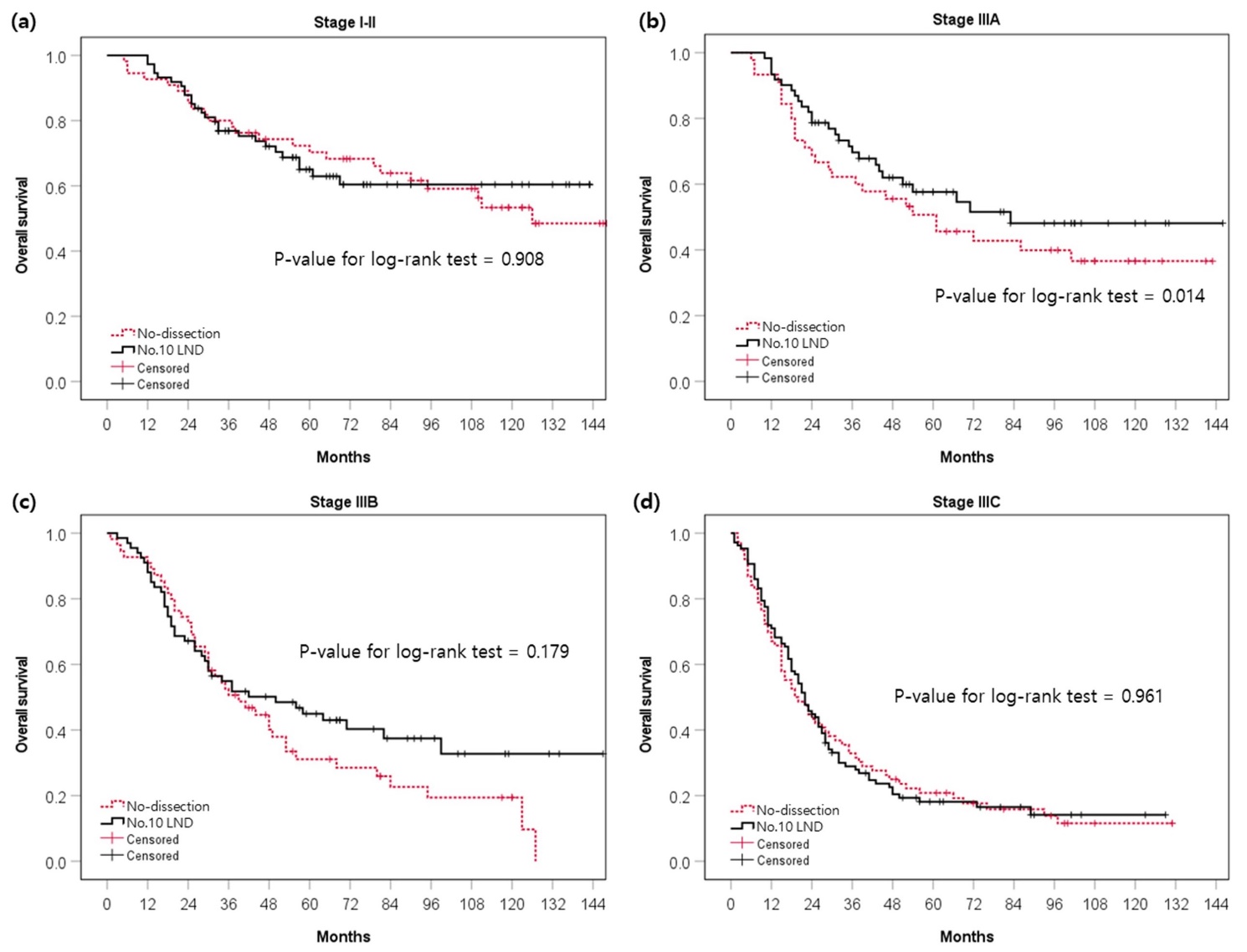

Supplement: Supplementary file 1 — Supplementary Figures. [file 41598_2023_42707_MOESM1_ESM.docx]
